# Understanding the Hedgehog Signaling Pathway in Acute Myeloid Leukemia Stem Cells: A Necessary Step toward a Cure

**DOI:** 10.3390/biology10040255

**Published:** 2021-03-24

**Authors:** Daniel Lainez-González, Juana Serrano-López, Juan Manuel Alonso-Domínguez

**Affiliations:** 1 Experimental Hematology, Instituto de Investigación Sanitaria Fundación Jiménez Díaz, Universidad Autónoma de Madrid, Avenida Reyes Católicos 2, 28040 Madrid, Spain; daniel.lainez@fjd.es (D.L.-G.); juana.serrano@quironsalud.es (J.S.-L.); 2 Hematology Department, Hospital Universitario Fundación Jiménez Díaz, Avenida Reyes Católicos 2, 28040 Madrid, Spain

**Keywords:** Hedgehog, quiescence, acute myeloid leukemia (AML), leukemic stem cell, crosstalk

## Abstract

**Simple Summary:**

The Hedgehog signaling pathway is related to the cell cycle. In particular, it is considered to play a fundamental role in the quiescence of leukemic stem cell (i.e., a temporary resting state without cell replication). Leukemic stem cells are the cells supposed to give rise to the relapses of the leukemia. Therefore, the Hedgehog pathway must be understood to improve the current treatments against acute myeloid leukemia and avoid the relapse of the disease. In this review, we gather the present knowledge about the physiological Hedgehog pathway function, the aberrant activation of Hedgehog in leukemia, and highlight the lack of evidence regarding some aspects of this important pathway. Finally, we summarize the acute myeloid leukemia treatments targeting this signaling pathway.

**Abstract:**

A better understanding of how signaling pathways govern cell fate is fundamental to advances in cancer development and treatment. The initialization of different tumors and their maintenance are caused by the deregulation of different signaling pathways and cancer stem cell maintenance. Quiescent stem cells are resistant to conventional chemotherapeutic treatments and, consequently, are responsible for disease relapse. In this review we focus on the conserved Hedgehog (Hh) signaling pathway which is involved in regulating the cell cycle of hematopoietic and leukemic stem cells. Thus, we examine the role of the Hh signaling pathway in normal and leukemic stem cells and dissect its role in acute myeloid leukemia. We explain not only the connection between illness and the signaling pathway but also evaluate innovative therapeutic approaches that could affect the outcome of patients with acute myeloid leukemia. We found that many aspects of the Hedgehog signaling pathway remain unknown. The role of Hh has only been proven in embryo and hematopoietic stem cell development. Further research is needed to elucidate the role of GLI transcription factors for therapeutic targeting. Glasdegib, an SMO inhibitor, has shown clinical activity in acute myeloid leukemia; however, its mechanism of action is not clear.

## 1. Hedgehog Signaling Pathway 

It is important to understand the interactions between the main Hedgehog (Hh) components to understand its role in cancer and how to effectively target these components to achieve or facilitate cancer cure. Our objective with this review is to compile and clarify the current evidence regarding the Hh pathway and encourage the research community to shed light on those aspects of this crucial pathway that remain unclear.

The Hh signaling pathway was described 40 years ago in *Drosophila melanogaster* by Nüsllein et al. They discovered three important proteins: cubitus interruptus (CI), hedgehog (HH), and patched (PTC) [1]. This pathway plays a major role in organism development through the regulation of single-cell fate and is highly conserved among species which highlights its importance across the evolutionary chain [2,3]. Nonetheless, some differences should be explained. In *D. melanogaster*, the signal is transmitted through the protein complex costal 2 (COS2), fused (FU), and suppressor of FU (SUFU) ending in CI, the transcription factor. In superior organisms such as mammals, FU and COS2 are not conserved, and the transcription factor is called GLI, from glioma, instead of CI [4]. 

In mammals, the Hh pathway is activated due to the paracrine signaling of the extracellular proteins sonic hedgehog (SHH), desert hedgehog (DHH), or Indian hedgehog (IHH). Hh protein release is mediated by Dispatched1 (DISP1) and SCUBE2 [5]. The role of SCUBE2 is fundamental in this process to overcome the insolubility conferred by SHH cholesterol modification [5]. SHH, DHH and IHH act as ligands for the 12-pass transmembrane receptor PTC which regulates the translocation to the primary cilium of the seven-pass transmembrane protein Smoothed (SMO). The presence of SMO in the cilium modifies the function of the zinc-finger transcription factors GLI1, GLI2, and GLI3 [4]. Therefore, the function of the primary cilium for canonical activation is essential. In fact, the primary cilia is present on 95–97% of peripheral blood and bone marrow cells [6]. Indeed, several authors correlate the response of some signaling pathways as Hh with the presence of the primary cilia [7,8]. Regarding GLI proteins, whereas GLI1 seems to have a minor role by amplifying the transcriptional response [9], GLI2 and GLI3 switch between activator (GLIA) or repressor (GLIR) forms. Moreover, GLI2 is a major pathway activator [10]. Furthermore, the active form of GLI2/GLI3 could elevate the expression of GLI1, which simultaneously amplifies the transcriptional response [11,12,13]. Finally, two PTC proteins are described: *PTC1* seems to be a tumor suppressor gene, and *PTC2*, although it is rarely mentioned, seems to have similar functions to *PTC1* [14]. Whilst PTC seems to be the main receptor for this signaling pathway, a diverse group of proteins act as co-receptors. Tenzen et al. suggested that the binding between CDO (CAM-related/downregulated by oncogenes) or BOC (Brother of CDO) with SHH either facilitating the presentation of the active ligand to PTC1 or increasing the effective levels of signaling in a responding cell [15]. Furthermore, GAS1 (the protein of Growth Arrest-Specific Gene 1) seems to activate Hh signaling [16]. 

**Figure 1:**
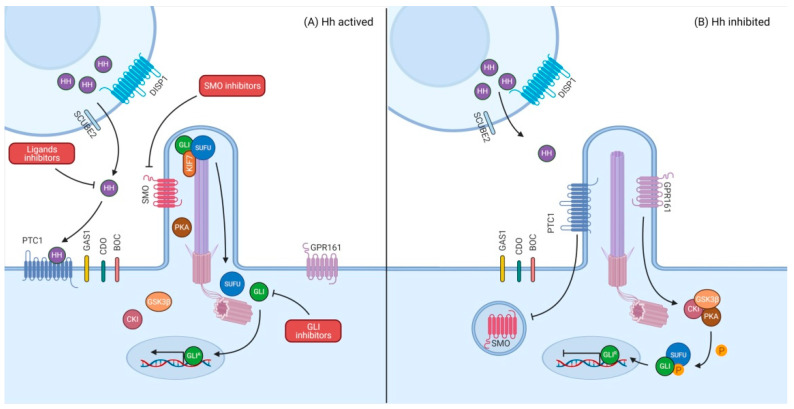
Hedgehog signaling pathway and its possible inhibitions. (**A**) HH ligands bind PTC1. GLI is dislocated from SUFU due to the translocation of SMO to the primary cilium. Promoters of target genes are triggered by the entrance of an active form of GLI into the nucleus. The signaling can be inhibited by targeting the ligands SHH, IHH or DHH, SMO or GLI. (**B**) PTC1 acts as a negative modulator of SMO. GLI is truncated in the base of the primary cilium by a protein kinases group giving a repressor form of GLI which inhibit the transcription of target genes.

Due to the binding of the HH ligands over the negative modulator PTC1, SMO is not inhibited and translocates into the primary cilium, which results in PTC1 and the seven-pass transmembrane G-protein coupled receptor 161 (GPR161) exiting the cilia. GPR161 does not activate protein kinase A (PKA) which is the major negative regulator of the pathway. When the complex SUFU-GLI2/GLI3 is promoted to the tip of the cilium by the kinesin-like protein KIF7, it is disassociated [10,17,18]. Subsequently, GLI2/GLI3 (GLIA) enters the nucleus and activates the transcription targets such as cellular proliferation genes (*MYCN*, *CCND1*, *CCND2*), cell fate genes (*FOXA2*, *FOXC2*, *SOX12*, or TBX2), and death cell regulation genes (*FAS*, *DR4*, *DR5*) (Figure 1A) [10,17,19,20,21,22,23]. 

On the other hand, the inhibition of this pathway occurs when HH ligands are not bound to PTC1 and, therefore, SMO is retained outside the primary cilium. GPR161 activates PKA which, alongside glycogen synthase kinase 3β (GSK3β) and casein kinase 1 (CK1), produce different patterns of phosphorylation on full length GLI2/GLI3 at the base of the cilium. This is followed by partial degradation through proteasome to generate the repressor form of GLI (GLIR) that represses the expression of some Hh target genes (Figure 1B) [10,17]. 

In this review we put the spotlight on the role of Hedgehog, but other signaling pathways are involved with Hedgehog regulation signaling. There are many authors who study the cross-talk between Hedgehog and other related pathways, such as Wnt, Notch, Hox, or mTOR [24,25]. The list of pathways which can interact with Hedgehog is large, but the most known cross-talk is performed by Wnt and Notch [26,27]. Indeed, these three pathways seem to have an indispensable role in stem cell (SC) maintenance and self-renewal capacity [28,29,30,31].

## 2. Hedgehog Signaling Activation in Cancer 

Cancer growth is driven by the disruption of molecular networks that control multicellularity. Hh signaling is no exception, and several alterations in this pathway have been found in different types of cancer [32]. In 1996, two important works were published describing *PTC1* mutations in patients with Gorlin syndrome, a condition that increases the risk of developing tumors, among other diseases [33,34]. This finding shed light on the possible association of Hh signaling with cancer. 

**Figure 2:**
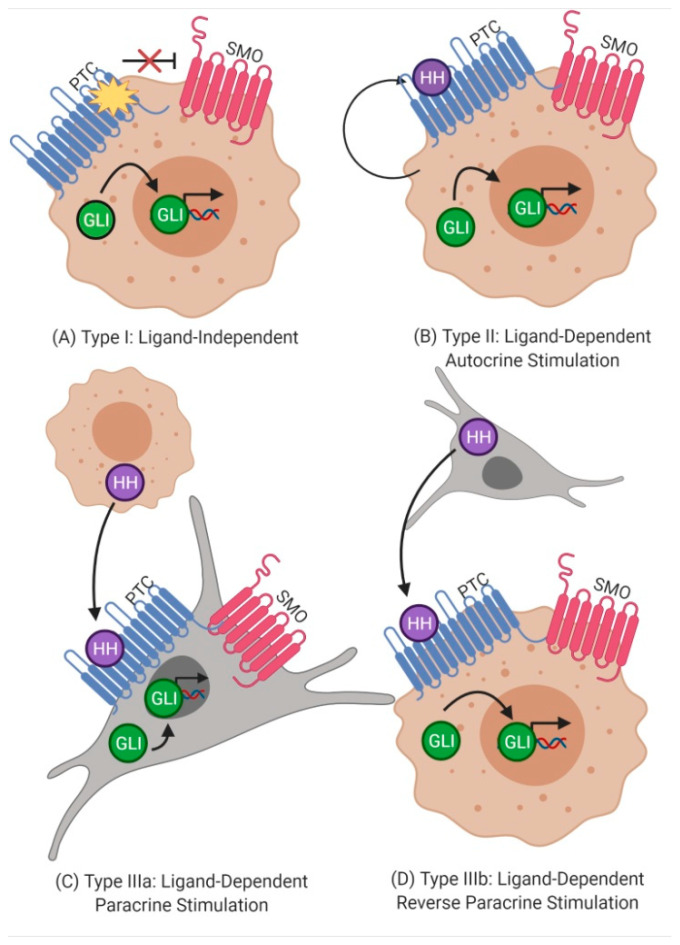
Models of signaling in Hedgehog pathway activation in cancer. (**A**) Type I: Ligand-Independent. (**B**) Type II: Ligand-Dependent Autocrine Stimulation. (**C**) Type III: Ligand-Dependent Paracrine Stimulation. (**D**) Type III: Ligand-Dependent Reverse Paracrine Stimulation.

The overexpression of the Hh pathway eventually leads to increased expression of target genes [35]. This overexpression may be due to a mutation or ligand expression. To understand ligand expression, three models of Hh pathway activation in cancer have been described. The first model is called Type I ligand-independent, in which tumor cells exhibit some mutations in the Hh pathway that encourage cell survival and growth. Loss-of-function mutations in PTCH1 activate SMO rendering an accumulation of GLI in the nucleus that promotes the transcription of target genes (Figure 2A). The second model is known as Type II model activation is ligand-dependent autocrine stimulation. In this model, the Hh ligand is self-secreted. The tumor cell expresses the protein HH which binds to PTCH1 and activates SMO (Figure 2B). The last model can be split into two paracrine stimulation models. In Type IIIa ligand-dependent paracrine stimulation, the tumor cell generates the Hh ligand, and Hh signaling is activated in an adjacent stroma cell. This creates a favorable microenvironment for tumor growth (Figure 2C). On the contrary, Type IIIb consists of Hh secretion by the stroma cell and subsequent activation of the tumor cell. The latter is known as ligand-dependent reverse paracrine stimulation (Figure 2D) [21,36].

The deregulation of Hh signaling may originate cancer in those organs in which this signaling pathway has a role in SC renewal [37,38,39,40,41,42,43,44,45,46,47,48,49]. Physiologically, SCs have the potential of either self-renewal (symmetric division) or differentiation (asymmetric division). Both processes give rise at least to a new SC, which renders SCs inexhaustible, and are strictly regulated so that the division of SCs is less frequent than that of regular cells. Therefore, SCs are usually in a quiescent state.

It is appropriate to recall that the concept of SCs was considered nearly 60 years ago [50], and it was established in acute myeloid leukemia (AML) as leukemic SCs (LSCs) by Dick’s group [51]. Afterward, the concept was extended to other types of cancer such as cancer SCs (CSCs) which have the same capacities as SCs.

## 3. Hedgehog Signaling in Normal Hematopoiesis

The role of the Hh signaling pathway in hematopoietic SCs (HSCs) has remained controversial. The existence of HSCs was proven during a study in which they were able to repopulate the hematological lineages in transplanted mice [52]. 

In the Hh pathway, SMO is usually selected in studies as it is a unique, non-redundant component of the pathway. The inhibition of SMO via inducible genetic suppression in a *Cre-LoxP* murine model highlights the dispensable role of this signaling pathway in adult HSCs; at 4 weeks post-treatment, there was no alteration in the absolute number of HSCs, defined as Lin^-^Sca1^+^cKit^+^ (LSK). Therefore, *Smo* knockout seems to have no effect on the expansion of the HSC compartment. Furthermore, these cells differentiate adequately into hematopoietic, namely myeloid, progenitors. On the other hand, this finding could be explained due to the redundancy between Notch, another of the highly conserved cellular signaling pathways, and Hh in hematopoiesis. Nevertheless, based on experiments, neither Hh nor Notch appears to be redundant in governing HSC fate. Therefore, the downregulation of *Smo* does not appear to cause any significant variation in HSC behavior in mice models [53]. Similar results were obtained by Hofmann et al. when they inhibited *Smo* in two different manners, genetically and pharmacologically. They demonstrated that the inhibition of Hh signaling does not affect the homeostasis of hematopoietic cells in adult mice [54]. The results from these two studies suggest that the inhibition of Hh signaling does not affect normal hematopoiesis. 

Despite the apparent lack of importance of the Hh pathway in hematopoiesis, other studies defend its role in HSCs. In 2005, Gering et al. showed that the Hh pathway is required in embryonic zebrafish to form the hematologic progenitors [55]. It should be noted that this study was conducted in a zebrafish model, a very different species from mammals. Nevertheless, similar outcomes have been found in mice model studies. The depletion of *Smo* in chimeric mice has essential consequences for renovating the HSCs compartment since a deficiency in the population of long-term HSCs (LT-HSCs) was seen when primary and secondary transplants were performed [56]. In this study, a non-conditional allele, *Vav-Cre*, caused a malformation in embryo development, which may have some repercussions in the adult mice. However, in Hofmann’s study, *Smo* loss was induced in mice in adulthood verifying that there was no deregulation during the embryonic phase [54]. 

Therefore, the role of Hh during adult HSC maintenance is unclear. However, it seems that all the studies draw the same conclusion regarding HSC development: Hh is essential for correct HSC development and correct formation of the whole embryo [29]. It is important to highlight that Hh seems to play a major role in HSC quiescence regulation as was shown by Trowbridge et al. The Hh downstream signaling pathway is involved in controlling HSC cycling and expansion during acute hematopoiesis recovery. By studying *Ptc1*^+/^^−^ in a mouse model, Trowbridge et al. demonstrated which genes are intermediate players in Hh cell cycle regulation, suggesting that an active role of Hh results in a greater number of cells in the cell cycle. Therefore, they proposed that continuous activation of Hh exhausts HSCs. Nevertheless, these experiments did not show a relationship between Hh and leukemogenesis since HSCs were not transformed into LSCs [57]. Consequently, this pathway may be important for the maintenance of AML but not for its formation [53,54,58]. 

As previously explained, there are models in which Hh is activated by soluble ligands as a paracrine or autocrine stimulation. Therefore microenvironment may play a fundamental role in Hh activation. Ligands of the Hh pathway are expressed by bone marrow stromal cells and have been shown to function as survival signals for lymphoid and myeloid malignancies [59]. Moreover, supernatant enriched with human mesenchymal stem cell-derived exosomes, isolated from in vitro assay, activated tumor growth depending on Hh signaling pathway suggesting that non-hematopoietic cells from the niche can influence tumor survival [60]. However, the cell-extrinsic effect of Hh is less clear in adult HSC regarding the discrepancies from the different mice models, stress conditions, and scarce human evidence.

## 4. The Role of Hedgehog in Other Hematologic Neoplasia

Although this review focuses on AML, it is interesting to note that the Hh pathway has been associated with the pathogenesis of other hematologic neoplasia [61,62] such as chronic myeloid leukemia (CML) and acute lymphoblastic leukemia (ALL). Regarding ALL, either T-ALL or B-ALL, Hh seems to stimulate tumor growth since its inhibition abrogates tumor expansion [63,64,65]. CML is characterized by the reciprocal chromosomal translocation t(9;22)(q34;q11) that gives rise to the Philadelphia chromosome [66]. This translocation produces the *BCR-ABL* fusion gene, of which the chimeric protein shows deregulated tyrosine kinase activity. Targeting of the *BCR-ABL* fusion protein by tyrosine kinase inhibitors (TKIs) produces an adequate response in most cases [67]. Moreover, some patients with sustained deep molecular responses who discontinue TKI treatment show no signs of disease reappearance. Despite these promising results, approximately 50% of these patients relapse after treatment discontinuation [68]. This phenomenon is thought to be caused by the persistence of LSCs, and the Hh pathway appears to play an important role in LSC maintenance [69]. As a matter of fact, several studies have demonstrated that the inhibition of *Smo* possibly led to a reduction of the LSC compartment in CML [69,70]. As an example, Zhao et al. showed that the Hh pathway controls the frequency and maintenance of CML blast crisis LSCs [56]. In addition, the importance of this signaling pathway is tangible given that *PTC1* expression is useful for determining the response to the TKI, imatinib, in patients with CML [71,72].

## 5. The Role of Hedgehog in AML

This review focuses on the role of Hh in AML, specifically in LSCs. Dick’s team described the LSC concept in AML by its engraftment in non-obese diabetic mice with severe combined immunodeficiency disease (NOD/SCID mice) [51]. Functional analyses of LSCs by Dick’s laboratory resulted in the postulation that AML presents a hierarchical organization similar to hematopoiesis. Furthermore, they observed that human LSCs transplanted in a mouse model had different self-renewal capacities [51,73] demonstrating the heterogeneity of LSCs in AML. This evidence suggests that these LSCs derive from normal HSCs due to their heterogeneity [51,73,74]. However, genetic evidence has revealed that malignant transformation may also affect hematopoietic progenitors which acquire LSC properties [75]. Goardon et al. demonstrated that LSC capacity is found in the CD34^+^CD38^−^ and the more differentiated CD34^+^CD38^+^ compartments. Nevertheless, only the CD34^+^CD38^−^ fraction could give rise to the more differentiated progeny and not the other way around [76]. This finding supports the theory that LSCs can also be found in the more differentiated progenitors and highlights the hierarchy of AML. 

In addition, Hh signaling from the niche seems to be loss meaning since only cell autonomous effect can contribute to AML maintenance [69]. This possibility is endorsed for experiments in MLL-AF9 mouse model, in which normal hematopoiesis is removed during AML as a consequence of hematopoietic niche cells depletion by AML blasts [77].

Since LSC persistence appears to be the source of the high relapse rate in AML, different groups have attempted to eradicate this population by targeting Hh. Hh inhibition appears to force LSCs to enter the cell cycle resulting in greater sensitivity to conventional chemotherapy [78,79]. 

On the other hand, the inhibition of this pathway is not trivial. There are three main possibilities to target Hh: its ligands, SMO or GLI. As previously explained, Hh seems to need primary cilia to exert its function and hematopoietic cells normally posses them. Nevertheless, Sing et al. described that the primary cilia were only present on 10–36% of leukemic cells [6]. This fact entails that Hh activation in AML may occur aberrantly [6]. Therefore, the study of downstream proteins such as GLI could be interesting, since they are the last player on cascade.

Chaudhry et al. studied the relationship between the components of the Hh pathway in AML. They described an important inverse correlation between GLI1 and GLI3^R^. The group demonstrated that GLI3 is epigenetically silenced in most AMLs, which leads to an activation of the target gene due to the lack of GLI3^R^. They suggested that the Hh pathway is active because of the loss of GLI3^R^ rather than SMO-based activation [80]. Wellbrock et al. also studied the role of GLI proteins in AML. GLI1 and GLI2 expression had a negative impact on patients outcomes. It was observed that GLI3 was absent in 74% of AML patient samples. Moreover, they found that there was no expression of HH ligands in their cohort [81]. Recently, the group suggested that the downregulation of GLI3 may be associated with a low sensitivity to cytarabine (Ara-C) [82], which may indicate that low levels of this protein protect LSCs or progenitor cells from the effect of Ara-C. Furthermore, other studies that targeted the refractory/relapse population in AML have seen that overexpression of GLI1 leads to an increase in drug resistance [83]. The elevation of GLI1 in cells resistant to Ara-C has been studied; high GLI1 expression occurs in refractory patients with rapid and repeated relapses [84]. Sensitivity may be restored by inhibiting or regulating the Hh signaling pathway [85]. This was confirmed by several reports, including one by Liang et al. who demonstrated that the inhibition of GLI1 increased Ara-C sensitivity in primary samples of AML [83].

## 6. Hedgehog Signaling Pathway as a Therapeutic Target in AML

Currently, patients with AML who are fit for treatment are mainly treated with chemotherapeutics agents such as Ara-C and anthracyclines. The standard AML induction therapy is known as 3+7 (i.e., three-day infusion of anthracyclines and a concomitant seven-day infusion of Ara-C). Following the induction cycle, a variable number of Ara-C-containing cycles are administered with or without a subsequent allogenic bone marrow transplantation, depending on the genetic risk of the disease [86]. Older or unfit patients have a worse prognosis due to the impossibility of receiving high doses of chemotherapy alongside their worse molecular profile [87]. The treatment of unfit patients is based on Ara-C administered at lower doses than in fit patients, or hypomethylating agents (i.e., Decitabine or Azacytidine [Aza]) [86]. Different molecules, mainly targeted therapies, are under development for this disease and have been approved in recent years in combination with any of the backbone AML therapies [88]. In 2018, Glasdegib became the first SMO inhibitor approved by the Food and Drug Administration for patients ≥75 years old or patients with preexisting comorbidities aged ≥55 years in whom intensive chemotherapy is not recommended [89]. Nevertheless, SMO is not the only targetable component of the Hh pathway. The effectors of the pathway (i.e., GLI transcription factors) can also be inhibited. Given the lack of clinical and preclinical evidence regarding HH ligand inhibition in AML, this treatments has not been addressed.

### 6.1. Targeting SMO

Cyclopamine, the first SMO inhibitor, was described in 1963 [90]. This alkaloid was demonstrated to inhibit SMO in vitro and in vivo. However, clinical trials were stopped due to several complications [91]. As previously explained, the only approved therapy targeting Hh in AML is Glasdegib, an SMO inhibitor. We will first consider other SMO inhibitors tested in AML for which clinical development has stopped due to an apparent lack of efficacy (Table 1).

Vismodegib is the first selective Hh inhibitor approved for the treatment of locally advanced and metastatic basal cell carcinoma (BCC) [92]. A phase Ib trial of vismodegib showed minimal clinical efficacy in patients with relapsed AML suggesting that this drug is not a good choice for AML treatment [93]. Sonidegib is another approved drug for BCC treatment [94]. Sonidegib in combination with Aza produces a synergetic effect in vitro and ex vivo in some primary AML patient samples [95]. Huang et al. also demonstrated that the combination of sonidegib and doxorubicin in xenograft mice leads to significant tumor regression. Besides, experiments performed in cell lines and refractory primary AML samples exhibited lower resistance to doxorubicin and cell apoptosis induction [84]. A phase I/Ib study of sonidegib in combination with Aza demonstrated that this combination was safe for patients. Nevertheless, remission rates are similar to single-drug treatment with Aza in pre-treated and relapsed/refractory AML patients [84,96,97]. Based on these results, neither sonidegib nor vismodegib entered into advanced phase clinical trials in AML. 

Glasdegib in combination with low-dose Ara-C (LDAC) has been demonstrated to increase overall survival (median of 8.8 vs. 4.9 months) in a phase 2 randomized study with Ara-C as the control therapy [98]. Nonetheless, some adverse effects were recorded. The most common serious adverse reactions in patients receiving glasdegib with LDAC were febrile neutropenia, pneumonia, hemorrhage, anemia and sepsis [98]. 

Glasdegib is hypothesized to enhance the sensitivity of dormant LSCs to chemotherapeutic agents [78,79]. Glasdegib does not affect SMO levels; however, it blocks the SMO translocation to cilia [80]. Currently, glasdegib combined with Aza and 3+7 for unfit and fit patients, respectively, is being explored in the BRIGHT AML1019 trial [99]. More attention has been paid to the combination of venetoclax plus azacitidine in older or unfit AML patients, probably due to the confirmation of its results in a phase 3 clinical trial. Nevertheless, the prognosis of AML patients remains dismal. The combination of different targeted drugs and/or the discovery of predictor markers of response to targeted therapies may help to improve clinical outcome of AML patients.

### 6.2. Targeting GLI

There are no clinical trials regarding GLI inhibition. Nevertheless, some groups have preliminary results of this inhibition in AML cell lines and primary cells. Latuske et al. studied six different cell lines and discovered that SMO is mostly not needed in the GLI signaling cascade in AML cell lines because the treatment with an SMO inhibitor, specifically cyclopamine, had barely any effects on GLI. In contrast, using a GLI inhibitor, namely GANT61, resulted in decreased GLI activity [100]. This could be explained as a non-canonical activation of Hh in those cell lines. On the other hand, a different option could be to restore the expression of GLI3^R^. Wellbrock et al. used GANT61 and observed how this treatment led to apoptosis, growth reduction, and decreased colony formation in vitro in patients with AML [81].

## 7. Discussion

The conservation of Hh among species highlights the importance of this pathway [2]. Hh is essential for cellular development and has a fundamental role in some cancers [37,38,39,40,41,42,43,44,45,46,47,48,49]. Three models of Hh activation have been described: ligand-independent, ligand-dependent autocrine stimulation, and ligand-dependent paracrine stimulation. Some types of stimulation suggest that the microenvironment has a significant function in Hh regulation [21,36].

The role of Hh in normal hematopoiesis is not clear. Nevertheless, all studies agree that Hh is crucial for the correct development of the embryo and HSC development. Further studies about its importance in HSC maintenance are needed to understand all the possible repercussions of drugs that inhibit Hh [53,54,58]. So far, given the apparent lack of importance of Hh in normal adult HSC, there seems to be a therapeutic window to target Hh, preserving normal hematopoiesis. 

CML is the paradigm for the molecular characterization and targeting of a tumoral disease. From our perspective, CML should once again serve as a guide as it highlights the importance of LSC eradication to avoid relapse of AML.

Hh appears to play an important role in the quiescence regulation of HSCs and LSCs of AML. However, evidence about this topic is contradictory. Some authors explain that Hh inhibition reduces LSC cycling, while others state that the same process results in LSC activation [78,79]. The role of Hh must be elucidated to comprehend which approach is better: its inhibition or activation. It is difficult to understand the mechanisms underlying these contradictory results. A possible explanation could be that LSCs do not behave like normal HSCs. Indeed, LSCs do not appear to be the malignant counterpart of HSCs and can also derive from differentiated progenitors that acquire self-renewal capacities. The main marker that must be used in flow cytometry to characterize AML-LSCs is CD34^+^CD38^-^. Studies that suggest that glasdegib targets dormant LSCs [78,79] based their results on a more general population, CD45^dim^. This approach is not correct since the cell cycle is being studied not only in LSCs but also in progenitors and more differentiated populations. If glasdegib does not affect LSC quiescence, its clinical effect could be explained by evidence that different components of the Hh pathway are upregulated in patients with AML with worse outcomes. A possible explanation is that in AML, the overexpression of GLI1 entails an increase in drug resistance as mentioned in Section 5 [83]. To summarize, more studies must be performed in order to clarify the mechanism of action of glasdegib. In case glasdegib inhibits LSC quiescence, conventional chemotherapy could reduce or eradicate this cell population. In this way we could decrease the relapses of the disease and increase survival of AML patients.

Although Wellbrock suggests that the absence of HH ligands could be due to a paracrine interaction between leukemic and bone marrow stromal cells [81] the role of non-canonical activation cannot be forgotten. Regarding this topic, we questioned whether GLI inhibition instead of SMO targeting results in more effective Hh inhibition. Thus far, only in vitro findings support this hypothesis; however, clinical evidence in patients with AML is required to test its plausibility. Therefore, the exact roles of the three GLI transcription factors should be elucidated urgently since it is unclear in both the physiological and tumoral setting. The best targets for this pathway are possibly these proteins since they are downstream of SMO. By targeting GLI, the way that Hh is activated, canonical or non-canonical, is not important. SMO could be inhibited; however, non-canonical activation may be able to generate GLI^A^. One way to target GLI proteins could be by the activation of GLI3^R^ using hypomethylating agents [80]. Additionally, low levels of GLI3 result in a protective effect from the cytotoxic effects of Ara-C on LSCs or progenitors cells [82].

Along this review, we have studied the Hh signaling pathway focusing on its canonical activation. Hh also interacts with a variety of signaling pathways such as Notch and/or Wnt, among other pathways [29,101,102]. Therefore, it might not be sufficient to inhibit only Hh and combination strategies targeting these CSC-related pathways can be an interesting approach [103].

## 8. Conclusions

It should be noted that we are ignorant of many aspects of the Hh pathway. Only the role of Hh in embryo and HSC development has been proven. The exact role of GLI transcription factors should be clarified prior to testing its role in therapeutic targeting at the clinical level. The inhibition of GLI^A^ or activation of GLI^R^ should be investigated since this complex is formed by the last proteins downstream; then, either Hh canonical activation or Hh non-canonical activation could be controlled. 

At present, the only drug approved for Hh, glasdegib, has shown clinical activity in AML; however, its mechanism of action is not clear due to contradictory in vitro results regarding the Hh quiescence regulation of LSC. 

Currently, we are studying the mechanism of action of glasdegib to understand the role of Hh in LSCs. The optimal way to improve the research not only regarding this topic but in science in general is by collaboration instead of competition. Collaborations with other research groups and correspondence regarding this review are welcomed. 

## Figures and Tables

**Table 1:** Clinical trial based on the inhibition of Hedgehog in acute myeloid leukemia.

Drug	Mechanism of Action	Clinical Trial	Phase
Vismodegib	Direct binding to SMO	NCT03878524 *	I
		NCT01880437	Ib/II
		NCT02073838	II
Sonidegib	Direct binding to SMO	NCT02129101	Ib
		NCT01826214	II
Glasdegib	Direct binding to SMO	NCT04655391 *	Pilot/Ib
		NCT02038777 *	I
		NCT03390296 *	Ib/II
		NCT01546038	Ib/II
		NCT02367456 *	Ib
		NCT03226418 *	II
		NCT04051996 *	II
		NCT04231851 *	II
		NCT03416179 *	III
		NCT04168502 *	III
		NCT04093505 *	III

* Active clinical trials.

## References

[1] Nüsslein-Volhard C., Wieschaus E. (1980). Mutations Affecting Segment Number and Polarity in Drosophila. Nature.

[2] Goodrich L.V., Johnson R.L., Milenkovic L., McMahon J.A., Scott M.P. (1996). Conservation of the Hedgehog/Patched Signaling Pathway from Flies to Mice: Induction of a Mouse Patched Gene by Hedgehog. Genes Dev..

[3] Krauss S., Concordet J.P., Ingham P.W. (1993). A Functionally Conserved Homolog of the Drosophila Segment Polarity Gene Hh Is Expressed in Tissues with Polarizing Activity in Zebrafish Embryos. Cell.

[4] Bürglin T.R. (2008). The Hedgehog Protein Family. Genome Biol..

[5] Tukachinsky H., Kuzmickas R.P., Jao C.Y., Liu J., Salic A. (2012). Dispatched and Scube Mediate the Efficient Secretion of the Cholesterol-Modified Hedgehog Ligand. Cell Rep..

[6] Singh M., Chaudhry P., Merchant A.A. (2016). Primary Cilia Are Present on Human Blood and Bone Marrow Cells and Mediate Hedgehog Signaling. Exp. Hematol..

[7] Huangfu D., Anderson K.V. (2005). Cilia and Hedgehog Responsiveness in the Mouse. Proc. Natl. Acad. Sci. USA.

[8] Goetz S.C., Ocbina P.J.R., Anderson K.V. (2009). The Primary Cilium as a Hedgehog Signal Transduction Machine. Methods Cell Biol..

[9] Park H.L., Bai C., Platt K.A., Matise M.P., Beeghly A., Hui C.C., Nakashima M., Joyner A.L. (2000). Mouse Gli1 Mutants Are Viable but Have Defects in SHH Signaling in Combination with a Gli2 Mutation. Development.

[10] Gorojankina T. (2016). Hedgehog Signaling Pathway: A Novel Model and Molecular Mechanisms of Signal Transduction. Cell. Mol. Life Sci..

[11] Dai P., Akimaru H., Tanaka Y., Maekawa T., Nakafuku M., Ishii S. (1999). Sonic Hedgehog-Induced Activation of the Gli1 Promoter Is Mediated by GLI3. J. Biol. Chem..

[12] Ding Q., Motoyama J., Gasca S., Mo R., Sasaki H., Rossant J., Hui C.C. (1998). Diminished Sonic Hedgehog Signaling and Lack of Floor Plate Differentiation in Gli2 Mutant Mice. Development.

[13] Buttitta L., Mo R., Hui C.-C., Fan C.-M. (2003). Interplays of Gli2 and Gli3 and Their Requirement in Mediating Shh-Dependent Sclerotome Induction. Development.

[14] Klein C., Kuhn A., Kissel S., Follo M., Illert A.L., Pfeifer D., Pahl H.L., Oostendorp R.A.J., Duyster J., Dierks C. (2015). Depletion of Ptch2 Activates Canonical and Non-Canonical HH Signaling within the Niche Leading to Myeloproliferation, Stem Cell Exhaustion and Accelerates JAK2V617F Driven Disease. Blood.

[15] Tenzen T., Allen B.L., Cole F., Kang J.-S., Krauss R.S., McMahon A.P. (2006). The Cell Surface Membrane Proteins Cdo and Boc Are Components and Targets of the Hedgehog Signaling Pathway and Feedback Network in Mice. Dev. Cell.

[16] Martinelli D.C., Fan C.-M. (2007). Gas1 Extends the Range of Hedgehog Action by Facilitating Its Signaling. Genes Dev..

[17] Briscoe J., Thérond P.P. (2013). The Mechanisms of Hedgehog Signalling and Its Roles in Development and Disease. Nat. Rev. Mol. Cell Biol..

[18] Hsu S.-H.C., Zhang X., Yu C., Li Z.J., Wunder J.S., Hui C.-C., Alman B.A. (2011). Kif7 Promotes Hedgehog Signaling in Growth Plate Chondrocytes by Restricting the Inhibitory Function of Sufu. Development.

[19] Katoh Y., Katoh M. (2009). Hedgehog Target Genes: Mechanisms of Carcinogenesis Induced by Aberrant Hedgehog Signaling Activation. Curr. Mol. Med..

[20] Katoh Y., Katoh M. (2008). Hedgehog Signaling, Epithelial-to-Mesenchymal Transition and MiRNA (Review). Int. J. Mol. Med..

[21] Rubin L.L., de Sauvage F.J. (2006). Targeting the Hedgehog Pathway in Cancer. Nat. Rev. Drug Discov..

[22] Kenney A.M., Rowitch D.H. (2000). Sonic Hedgehog Promotes G1 Cyclin Expression and Sustained Cell Cycle Progression in Mammalian Neuronal Precursors. Mol. Cell Biol..

[23] Duman-Scheel M., Weng L., Xin S., Du W. (2002). Hedgehog Regulates Cell Growth and Proliferation by Inducing Cyclin D and Cyclin E. Nature.

[24] Sengupta A., Banerjee D., Chandra S., Banerji S.K., Ghosh R., Roy R., Banerjee S. (2007). Deregulation and Cross Talk among Sonic Hedgehog, Wnt, Hox and Notch Signaling in Chronic Myeloid Leukemia Progression. Leukemia.

[25] Okuhashi Y., Itoh M., Tohda S. (2018). GLI1 and CTNNB1 Knockdown Activates NOTCH and MTOR Signalling in NB4 Myeloid Leukaemia Cells. Anticancer Res..

[26] Jacobs C.T., Huang P. (2019). Notch Signalling Maintains Hedgehog Responsiveness via a Gli-Dependent Mechanism during Spinal Cord Patterning in Zebrafish. Elife.

[27] Jacobs C.T., Huang P. (2021). Complex Crosstalk of Notch and Hedgehog Signalling during the Development of the Central Nervous System. Cell. Mol. Life Sci..

[28] Matsui W.H. (2016). Cancer Stem Cell Signaling Pathways. Medicine.

[29] Heidel F.H., Arreba-Tutusaus P., Armstrong S.A., Fischer T. (2015). Evolutionarily Conserved Signaling Pathways: Acting in the Shadows of Acute Myelogenous Leukemia’s Genetic Diversity. Clin. Cancer Res..

[30] Chatterjee S., Sil P.C. (2019). Targeting the Crosstalks of Wnt Pathway with Hedgehog and Notch for Cancer Therapy. Pharmacol. Res..

[31] Takebe N., Miele L., Harris P.J., Jeong W., Bando H., Kahn M., Yang S.X., Ivy S.P. (2015). Targeting Notch, Hedgehog, and Wnt Pathways in Cancer Stem Cells: Clinical Update. Nat. Rev. Clin. Oncol..

[32] Trigos A.S., Pearson R.B., Papenfuss A.T., Goode D.L. (2018). How the Evolution of Multicellularity Set the Stage for Cancer. Br. J. Cancer.

[33] Hahn H., Wicking C., Zaphiropoulous P.G., Gailani M.R., Shanley S., Chidambaram A., Vorechovsky I., Holmberg E., Unden A.B., Gillies S. (1996). Mutations of the Human Homolog of Drosophila Patched in the Nevoid Basal Cell Carcinoma Syndrome. Cell.

[34] Johnson R.L., Rothman A.L., Xie J., Goodrich L.V., Bare J.W., Bonifas J.M., Quinn A.G., Myers R.M., Cox D.R., Epstein E.H. (1996). Human Homolog of Patched, a Candidate Gene for the Basal Cell Nevus Syndrome. Science.

[35] Evangelista M., Tian H., de Sauvage F.J. (2006). The Hedgehog Signaling Pathway in Cancer. Clin. Cancer Res..

[36] Cochrane C.R., Szczepny A., Watkins D.N., Cain J.E. (2015). Hedgehog Signaling in the Maintenance of Cancer Stem Cells. Cancers.

[37] Beachy P.A., Karhadkar S.S., Berman D.M. (2004). Tissue Repair and Stem Cell Renewal in Carcinogenesis. Nature.

[38] Ruiz i Altaba A., Stecca B., Sánchez P. (2004). Hedgehog--Gli Signaling in Brain Tumors: Stem Cells and Paradevelopmental Programs in Cancer. Cancer Lett..

[39] Romer J.T., Kimura H., Magdaleno S., Sasai K., Fuller C., Baines H., Connelly M., Stewart C.F., Gould S., Rubin L.L. (2004). Suppression of the Shh Pathway Using a Small Molecule Inhibitor Eliminates Medulloblastoma in Ptc1(+/-)P53(-/-) Mice. Cancer Cell.

[40] Shahi M.H., Rey J.A., Castresana J.S. (2012). The Sonic Hedgehog-GLI1 Signaling Pathway in Brain Tumor Development. Expert Opin. Ther. Targets.

[41] Berman D.M., Karhadkar S.S., Maitra A., Montes De Oca R., Gerstenblith M.R., Briggs K., Parker A.R., Shimada Y., Eshleman J.R., Watkins D.N. (2003). Widespread Requirement for Hedgehog Ligand Stimulation in Growth of Digestive Tract Tumours. Nature.

[42] Van Dop W.A., Rosekrans S.L., Uhmann A., Jaks V., Offerhaus G.J.A., van den Bergh Weerman M.A., Kasper M., Heijmans J., Hardwick J.C.H., Verspaget H.W. (2013). Hedgehog Signalling Stimulates Precursor Cell Accumulation and Impairs Epithelial Maturation in the Murine Oesophagus. Gut.

[43] Konstantinou D., Bertaux-Skeirik N., Zavros Y. (2016). Hedgehog Signaling in the Stomach. Curr. Opin. Pharmacol..

[44] Li C., Heidt D.G., Dalerba P., Burant C.F., Zhang L., Adsay V., Wicha M., Clarke M.F., Simeone D.M. (2007). Identification of Pancreatic Cancer Stem Cells. Cancer Res..

[45] Watkins D.N., Berman D.M., Burkholder S.G., Wang B., Beachy P.A., Baylin S.B. (2003). Hedgehog Signalling within Airway Epithelial Progenitors and in Small-Cell Lung Cancer. Nature.

[46] Lim S., Lim S.M., Kim M.J., Park S.Y., Kim J.H. (2019). Sonic Hedgehog Pathway as the Prognostic Marker in Patients with Extensive Stage Small Cell Lung Cancer. Yonsei Med. J..

[47] Sanchez P., Hernández A.M., Stecca B., Kahler A.J., DeGueme A.M., Barrett A., Beyna M., Datta M.W., Datta S., Ruiz i Altaba A. (2004). Inhibition of Prostate Cancer Proliferation by Interference with SONIC HEDGEHOG-GLI1 Signaling. Proc. Natl. Acad. Sci. USA.

[48] Hamed S., LaRue H., Hovington H., Girard J., Jeannotte L., Latulippe E., Fradet Y. (2004). Accelerated Induction of Bladder Cancer in Patched Heterozygous Mutant Mice. Cancer Res..

[49] Kitagawa K., Shigemura K., Sung S.-Y., Chen K.-C., Huang C.-C., Chiang Y.-T., Liu M.-C., Huang T.-W., Yamamichi F., Shirakawa T. (2019). Possible Correlation of Sonic Hedgehog Signaling with Epithelial-Mesenchymal Transition in Muscle-Invasive Bladder Cancer Progression. J. Cancer Res. Clin. Oncol..

[50] Till J.E., McCulloch E.A. (1961). A Direct Measurement of the Radiation Sensitivity of Normal Mouse Bone Marrow Cells. Radiat. Res..

[51] Lapidot T., Sirard C., Vormoor J., Murdoch B., Hoang T., Caceres-Cortes J., Minden M., Paterson B., Caligiuri M.A., Dick J.E. (1994). A Cell Initiating Human Acute Myeloid Leukaemia after Transplantation into SCID Mice. Nature.

[52] Spangrude G.J., Heimfeld S., Weissman I.L. (1988). Purification and Characterization of Mouse Hematopoietic Stem Cells. Science.

[53] Gao J., Graves S., Koch U., Liu S., Jankovic V., Buonamici S., El Andaloussi A., Nimer S.D., Kee B.L., Taichman R. (2009). Hedgehog Signaling Is Dispensable for Adult Hematopoietic Stem Cell Function. Cell Stem Cell.

[54] Hofmann I., Stover E.H., Cullen D.E., Mao J., Morgan K.J., Lee B.H., Kharas M.G., Miller P.G., Cornejo M.G., Okabe R. (2009). Hedgehog Signaling Is Dispensable for Adult Murine Hematopoietic Stem Cell Function and Hematopoiesis. Cell Stem Cell.

[55] Gering M., Patient R. (2005). Hedgehog Signaling Is Required for Adult Blood Stem Cell Formation in Zebrafish Embryos. Dev. Cell.

[56] Zhao C., Chen A., Jamieson C.H., Fereshteh M., Abrahamsson A., Blum J., Kwon H.Y., Kim J., Chute J.P., Rizzieri D. (2009). Hedgehog Signalling Is Essential for Maintenance of Cancer Stem Cells in Myeloid Leukaemia. Nature.

[57] Trowbridge J.J., Scott M.P., Bhatia M. (2006). Hedgehog Modulates Cell Cycle Regulators in Stem Cells to Control Hematopoietic Regeneration. Proc. Natl. Acad. Sci. USA.

[58] Campbell V., Copland M. (2015). Hedgehog Signaling in Cancer Stem Cells: A Focus on Hematological Cancers. Stem Cells Cloning.

[59] Mar B.G., Amakye D., Aifantis I., Buonamici S. (2011). The Controversial Role of the Hedgehog Pathway in Normal and Malignant Hematopoiesis. Leukemia.

[60] Qi J., Zhou Y., Jiao Z., Wang X., Zhao Y., Li Y., Chen H., Yang L., Zhu H., Li Y. (2017). Exosomes Derived from Human Bone Marrow Mesenchymal Stem Cells Promote Tumor Growth Through Hedgehog Signaling Pathway. Cell Physiol. Biochem..

[61] Kobune M., Iyama S., Kikuchi S., Horiguchi H., Sato T., Murase K., Kawano Y., Takada K., Ono K., Kamihara Y. (2012). Stromal Cells Expressing Hedgehog-Interacting Protein Regulate the Proliferation of Myeloid Neoplasms. Blood Cancer J..

[62] Bai L.-Y., Chiu C.-F., Lin C.-W., Hsu N.-Y., Lin C.-L., Lo W.-J., Kao M.-C. (2008). Differential Expression of Sonic Hedgehog and Gli1 in Hematological Malignancies. Leukemia.

[63] Kawahara T., Kawaguchi-Ihara N., Okuhashi Y., Itoh M., Nara N., Tohda S. (2009). Cyclopamine and Quercetin Suppress the Growth of Leukemia and Lymphoma Cells. Anticancer Res..

[64] Ji Z., Mei F.C., Johnson B.H., Thompson E.B., Cheng X. (2007). Protein Kinase A, Not Epac, Suppresses Hedgehog Activity and Regulates Glucocorticoid Sensitivity in Acute Lymphoblastic Leukemia Cells. J. Biol. Chem..

[65] Lin T.L., Wang Q.H., Brown P., Peacock C., Merchant A.A., Brennan S., Jones E., McGovern K., Watkins D.N., Sakamoto K.M. (2010). Self-Renewal of Acute Lymphocytic Leukemia Cells Is Limited by the Hedgehog Pathway Inhibitors Cyclopamine and IPI-926. PLoS ONE.

[66] Nowell P.C. (1962). The Minute Chromosome (Phl) in Chronic Granulocytic Leukemia. Blut.

[67] Hochhaus A., Baccarani M., Silver R.T., Schiffer C., Apperley J.F., Cervantes F., Clark R.E., Cortes J.E., Deininger M.W., Guilhot F. (2020). European LeukemiaNet 2020 Recommendations for Treating Chronic Myeloid Leukemia. Leukemia.

[68] Saussele S., Richter J., Guilhot J., Gruber F.X., Hjorth-Hansen H., Almeida A., Janssen J.J.W.M., Mayer J., Koskenvesa P., Panayiotidis P. (2018). Discontinuation of Tyrosine Kinase Inhibitor Therapy in Chronic Myeloid Leukaemia (EURO-SKI): A Prespecified Interim Analysis of a Prospective, Multicentre, Non-Randomised, Trial. Lancet Oncol..

[69] Dierks C., Beigi R., Guo G.-R., Zirlik K., Stegert M.R., Manley P., Trussell C., Schmitt-Graeff A., Landwerlin K., Veelken H. (2008). Expansion of Bcr-Abl-Positive Leukemic Stem Cells Is Dependent on Hedgehog Pathway Activation. Cancer Cell.

[70] Babashah S., Sadeghizadeh M., Hajifathali A., Tavirani M.R., Zomorod M.S., Ghadiani M., Soleimani M. (2013). Targeting of the Signal Transducer Smo Links MicroRNA-326 to the Oncogenic Hedgehog Pathway in CD34+ CML Stem/Progenitor Cells. Int. J. Cancer.

[71] Alonso-Dominguez J.M., Grinfeld J., Alikian M., Marin D., Reid A., Daghistani M., Hedgley C., O’Brien S., Clark R.E., Apperley J. (2015). PTCH1 Expression at Diagnosis Predicts Imatinib Failure in Chronic Myeloid Leukaemia Patients in Chronic Phase. Am. J. Hematol..

[72] Alonso-Dominguez J.M., Casado L.F., Anguita E., Gomez-Casares M.T., Buño I., Ferrer-Marín F., Arenas A., Del Orbe R., Ayala R., Llamas P. (2017). PTCH1 Is a Reliable Marker for Predicting Imatinib Response in Chronic Myeloid Leukemia Patients in Chronic Phase. PLoS ONE.

[73] Hope K.J., Jin L., Dick J.E. (2004). Acute Myeloid Leukemia Originates from a Hierarchy of Leukemic Stem Cell Classes That Differ in Self-Renewal Capacity. Nat. Immunol..

[74] Bonnet D., Dick J.E. (1997). Human Acute Myeloid Leukemia Is Organized as a Hierarchy That Originates from a Primitive Hematopoietic Cell. Nat. Med..

[75] Chopra M., Bohlander S.K. (2019). The Cell of Origin and the Leukemia Stem Cell in Acute Myeloid Leukemia. Genes Chromosomes Cancer.

[76] Goardon N., Marchi E., Atzberger A., Quek L., Schuh A., Soneji S., Woll P., Mead A., Alford K.A., Rout R. (2011). Coexistence of LMPP-like and GMP-like Leukemia Stem Cells in Acute Myeloid Leukemia. Cancer Cell.

[77] Duarte D., Hawkins E.D., Akinduro O., Ang H., De Filippo K., Kong I.Y., Haltalli M., Ruivo N., Straszkowski L., Vervoort S.J. (2018). Inhibition of Endosteal Vascular Niche Remodeling Rescues Hematopoietic Stem Cell Loss in AML. Cell Stem Cell.

[78] Fukushima N., Minami Y., Kakiuchi S., Kuwatsuka Y., Hayakawa F., Jamieson C., Kiyoi H., Naoe T. (2016). Small-Molecule Hedgehog Inhibitor Attenuates the Leukemia-Initiation Potential of Acute Myeloid Leukemia Cells. Cancer Sci..

[79] Sadarangani A., Pineda G., Lennon K.M., Chun H.-J., Shih A., Schairer A.E., Court A.C., Goff D.J., Prashad S.L., Geron I. (2015). GLI2 Inhibition Abrogates Human Leukemia Stem Cell Dormancy. J. Transl. Med..

[80] Chaudhry P., Singh M., Triche T.J., Guzman M., Merchant A.A. (2017). GLI3 Repressor Determines Hedgehog Pathway Activation and Is Required for Response to SMO Antagonist Glasdegib in AML. Blood.

[81] Wellbrock J., Latuske E., Köhler J., Wagner K., Stamm H., Vettorazzi E., Vohwinkel G., Klokow M., Uibeleisen R., Ehm P. (2015). Expression of Hedgehog Pathway Mediator GLI Represents a Negative Prognostic Marker in Human Acute Myeloid Leukemia and Its Inhibition Exerts Antileukemic Effects. Clin. Cancer Res..

[82] Freisleben F., Behrmann L., Thaden V., Muschhammer J., Bokemeyer C., Fiedler W., Wellbrock J. (2020). Downregulation of GLI3 Expression Mediates Chemotherapy Resistance in Acute Myeloid Leukemia. Int. J. Mol. Sci..

[83] Liang H., Zheng Q.-L., Fang P., Zhang J., Zhang T., Liu W., Guo M., Robinson C.L., Chen S.-B., Chen X.-P. (2017). Targeting the PI3K/AKT Pathway via GLI1 Inhibition Enhanced the Drug Sensitivity of Acute Myeloid Leukemia Cells. Sci. Rep..

[84] Huang K., Sun Z., Ding B., Jiang X., Wang Z., Zhu Y., Meng F. (2019). Suppressing Hedgehog Signaling Reverses Drug Resistance of Refractory Acute Myeloid Leukemia. Onco Targets Ther..

[85] Zahreddine H.A., Culjkovic-Kraljacic B., Assouline S., Gendron P., Romeo A.A., Morris S.J., Cormack G., Jaquith J.B., Cerchietti L., Cocolakis E. (2014). The Sonic Hedgehog Factor GLI1 Imparts Drug Resistance through Inducible Glucuronidation. Nature.

[86] Döhner H., Estey E., Grimwade D., Amadori S., Appelbaum F.R., Büchner T., Dombret H., Ebert B.L., Fenaux P., Larson R.A. (2017). Diagnosis and Management of AML in Adults: 2017 ELN Recommendations from an International Expert Panel. Blood.

[87] Bullinger L., Döhner K., Döhner H. (2017). Genomics of Acute Myeloid Leukemia Diagnosis and Pathways. J. Clin. Oncol..

[88] Blum W.G., Mims A.S. (2020). Treating Acute Myeloid Leukemia in the Modern Era: A Primer. Cancer.

[89] Hoy S.M. (2019). Glasdegib: First Global Approval. Drugs.

[90] Binns W., James L.F., Shupe J.L., Everett G. (1963). A congenital cyclopian-type malformation in lambs induced by maternal ingestion of a range plant, veratrum californicum. Am. J. Vet. Res..

[91] Lin T.L., Matsui W. (2012). Hedgehog Pathway as a Drug Target: Smoothened Inhibitors in Development. Onco Targets Ther..

[92] Frampton J.E., Basset-Séguin N. (2018). Vismodegib: A Review in Advanced Basal Cell Carcinoma. Drugs.

[93] Bixby D., Noppeney R., Lin T.L., Cortes J., Krauter J., Yee K., Medeiros B.C., Krämer A., Assouline S., Fiedler W. (2019). Safety and Efficacy of Vismodegib in Relapsed/Refractory Acute Myeloid Leukaemia: Results of a Phase Ib Trial. Br. J. Haematol..

[94] Burness C.B. (2015). Sonidegib: First Global Approval. Drugs.

[95] Tibes R., Al-Kali A., Oliver G.R., Delman D.H., Hansen N., Bhagavatula K., Mohan J., Rakhshan F., Wood T., Foran J.M. (2015). The Hedgehog Pathway as Targetable Vulnerability with 5-Azacytidine in Myelodysplastic Syndrome and Acute Myeloid Leukemia. J. Hematol. Oncol..

[96] Tibes R., Kosiorek H.E., Dueck A.C., Sproat L., Palmer J., Slack J.L., Singh D., Gebhart E., Knight E., Hashmi S.K. (2015). Phase I/IB Study of Azacitidine and Hedgehog Pathway Inhibition in Myeloid Malignancies. Blood.

[97] Tibes R., Kosiorek H.E., Dueck A., Palmer J., Slack J.L., Knight E.A., Hashmi S.K., Bogenberger J.M., Zblewski D., Hogan W.J. (2017). Phase I/IB Study of Azacitidine and Hedgehog Pathway Inhibition with Sonidegib (LDE225) in Myeloid Malignancies. Blood.

[98] Cortes J.E., Heidel F.H., Hellmann A., Fiedler W., Smith B.D., Robak T., Montesinos P., Pollyea D.A., DesJardins P., Ottmann O. (2019). Randomized Comparison of Low Dose Cytarabine with or without Glasdegib in Patients with Newly Diagnosed Acute Myeloid Leukemia or High-Risk Myelodysplastic Syndrome. Leukemia.

[99] Cortes J.E., Dombret H., Merchant A., Tauchi T., DiRienzo C.G., Sleight B., Zhang X., Leip E.P., Shaik N., Bell T. (2019). Glasdegib plus Intensive/Nonintensive Chemotherapy in Untreated Acute Myeloid Leukemia: BRIGHT AML 1019 Phase III Trials. Future Oncol..

[100] Latuske E.-M., Stamm H., Klokow M., Vohwinkel G., Muschhammer J., Bokemeyer C., Jücker M., Kebenko M., Fiedler W., Wellbrock J. (2017). Combined Inhibition of GLI and FLT3 Signaling Leads to Effective Anti-Leukemic Effects in Human Acute Myeloid Leukemia. Oncotarget.

[101] Gu D., Xie J. (2015). Non-Canonical Hh Signaling in Cancer—Current Understanding and Future Directions. Cancers.

[102] Jenkins D. (2009). Hedgehog Signalling: Emerging Evidence for Non-Canonical Pathways. Cell. Signal..

[103] Stappenbeck F., Wang F., Tang L.-Y., Zhang Y.E., Parhami F. (2019). Inhibition of Non-Small Cell Lung Cancer Cells by Oxy210, an Oxysterol-Derivative That Antagonizes TGFβ and Hedgehog Signaling. Cells.

